# NK Cell Memory to Cytomegalovirus: Implications for Vaccine Development

**DOI:** 10.3390/vaccines8030394

**Published:** 2020-07-20

**Authors:** Calum Forrest, Ariane Gomes, Matthew Reeves, Victoria Male

**Affiliations:** 1 Institute of Immunity & Transplantation, UCL, Royal Free Campus, London NW3 2PF, UK; calum.forrest@ucl.ac.uk (C.F.); a.gomes@ucl.ac.uk (A.G.); 2 Department of Metabolism, Digestion and Reproduction, Imperial College London, Chelsea and Westminster Campus, London SW10 9NH, UK

**Keywords:** natural killer, NK, memory, HCMV, MCMV, cytomegalovirus

## Abstract

Natural killer (NK) cells are innate lymphoid cells that recognize and eliminate virally-infected and cancerous cells. Members of the innate immune system are not usually considered to mediate immune memory, but over the past decade evidence has emerged that NK cells can do this in several contexts. Of these, the best understood and most widely accepted is the response to cytomegaloviruses, with strong evidence for memory to murine cytomegalovirus (MCMV) and several lines of evidence suggesting that the same is likely to be true of human cytomegalovirus (HCMV). The importance of NK cells in the context of HCMV infection is underscored by the armory of NK immune evasion genes encoded by HCMV aimed at subverting the NK cell immune response. As such, ongoing studies that have utilized HCMV to investigate NK cell diversity and function have proven instructive. Here, we discuss our current understanding of NK cell memory to viral infection with a focus on the response to cytomegaloviruses. We will then discuss the implications that this will have for the development of a vaccine against HCMV with particular emphasis on how a strategy that can harness the innate immune system and NK cells could be crucial for the development of a vaccine against this high-priority pathogen.

## 1. Introduction

The immune response to infection is complex, involving multiple effector functions that act in a highly coordinated fashion. Importantly, the immune system can develop a memory of infection making it better able to respond to re-infection—a strategy that underpins the process of vaccination. Classically, this memory response is elicited by cell-mediated and humoral immunity. However, it is emerging that natural killer (NK) cells—usually considered innate immune cells—may also have the capacity for memory. 

One of the driving factors of the concept of NK memory has been the nature of the response to human cytomegalovirus (HCMV) infection. HCMV is a member of the herpesvirus family. It is a large, enveloped viruses with a double-stranded DNA (dsDNA) genome that establishes lifelong latent infection under constant immune surveillance, with occasional periods of reactivation [1]. Importantly, HCMV infection is common—with greater than 50% of young adults to more than 90% of the elderly infected. Developing countries typically have a higher incidence with more than 80% of young adults being seropositive [2,3].

Importantly, in healthy individuals HCMV infection is controlled by the prodigious immune response generated against it. In contrast, it is a major health burden in the immunocompromised and immune-immature. Transmission of the virus during pregnancy to the immunologically immature fetus can result in severe congenital disease. Around 1% of all newborns are congenitally infected with HCMV, with around 20% of these suffering from birth defects such as deafness and learning difficulties [4,5]. Additionally, HCMV poses a significant risk to immunosuppressed individuals, such as transplant and AIDS patients [6,7,8].

The significant public health and financial burden associated with HCMV disease, particularly among the congenitally infected, underpins the US National Institute of Medicine’s assertion that HCMV is a pathogen against which the development of a vaccine and novel treatment strategies are of the highest priority [9]. A number of immunization strategies have been trialed and have, logically, focused upon harnessing aspects of the adaptive immune response to natural infection with HCMV. Despite intensive research and a number of clinical studies demonstrating partial protection, no candidate vaccine has yet reached sufficient efficacy to be approved [10]. 

In this review, we will discuss the emerging concepts of NK cell memory and adaptive NK cell responses. In the light of recent findings that adaptive NK cells can control murine cytomegalovirus (MCMV), and the evidence that HCMV-driven NK cell memory also exists in humans, we will focus on the interaction of NK cells with HCMV to discuss the possibility of designing strategies to harness NK cells for the development of effective vaccines in the future. 

## 2. NK Cells 

Natural killer cells were first identified by their ability to kill cancerous target cells without the need for prior stimulation [11], although it later became apparent that they also have an important role in protecting the body against viruses, including cytomegalovirus (CMV) [12].

NK cells are members of the innate immune system and as such do not undergo somatic recombination of their antigen receptors. Instead, they express a variety of germline-encoded receptors that allow them to recognize their targets in several ways. The first of these to be described was “missing self” recognition, in which NK cells recognize targets that fail to express normal levels of major histocompatibility complex class I (MHC-I) [13]. This acts as a riposte to an immune evasion tactic common to viruses and cancer, in which MHC-I is downregulated in order to avoid recognition by CD8^+^ T cells. “Missing self” recognition is achieved by the expression of inhibitory receptors that recognize MHC-I, such as killer immunoglobulin-like receptors (KIRs) in humans or Ly49 molecules in mice. When MHC-I is absent, the inhibitory signal is not delivered, tipping the balance in favor of NK cell activation.

It has since come to be appreciated that NK cells can also be activated by the gain of an activating signal [14]. For example, the activating receptor NKG2D recognizes ligands whose expression is increased on stressed and cycling cells. NK cells can also be activated by antibody-coated targets, which they recognize via FcγRIII (CD16): NK cell killing of these targets is called antibody-dependent cell-mediated cytotoxicity (ADCC). Finally, they can also recognize certain virus-derived ligands directly. An instructive example is the murine NK cell receptor Ly49H, which is an activating version of the inhibitory receptor Ly49I. MCMV produces an MHC-I homologue, m157, which inhibits NK cell activation via the inhibitory receptor Ly49I. In some strains of mice, the Ly49I gene was duplicated and one copy converted to the activating version Ly49H: NK cells bearing Ly49H in these strains directly recognize and are activated by virally-infected cells expressing m157 [15]. Finally, NK cells can be activated by cytokines such as interleukin-15 (IL-15), IL-12 and IL-18. Following activation by any of these means, NK cells release cytotoxic granules which induce apoptosis in the target cell, as well as producing large amounts of interferon-γ (IFNγ).

NK cells account for approximately 10% of circulating immune cells in humans, but there are other tissues in which they are more frequent. In human liver, for example, 25–50% of total immune cells are NK cells [16]. In both humans and mice, approximately half of the NK cells in the liver are phenotypically and functionally similar to circulating NK cells and move freely between the liver and blood. The other half express proteins that are associated with tissue residency, are long-lived and unable to leave the liver [17]. In mice, these liver-resident NK cells are now accepted as belonging to a recently-defined family of immune cells that are closely related to NK cells, called type 1 innate lymphoid cells, or ILC1 [18]. In humans, it is not yet clear whether these cells more closely represent NK cells or ILC1: here we call them “liver-resident NK cells” in both species. Populations of NK cells that somewhat resemble liver-resident NK cells can also be found in the uterus and are particularly abundant in the first trimester of pregnancy [19]. Although not yet as well-understood as circulating NK cells, these tissue-resident populations may also have a role to play in viral defense.

## 3. NK Cell Memory

As members of the innate immune system, NK cells have traditionally been considered not to have memory, but over the past decade it has become clear that NK cells do, in fact, have some capacity to learn from experience. Three ways in which they can do this have so far been described: antigen-specific memory of liver-resident NK cells, cytokine-induced memory and CMV-driven memory.

### 3.1. Antigen-Specific Memory in Liver-Resident NK Cells

The first evidence that NK cells possess features of memory came from experiments showing that Rag^-/-^ mice, which lack T and B cells, display contact hypersensitivity to haptens, and that this can be abrogated by depleting NK cells [20]. Subsequent work showed that the relevant cells are liver-resident [21,22] and can also mediate protective responses to vaccines against influenza and VSV [21]. Inhibitory Ly49 receptors, which recognize specific peptides being presented on MHC-I molecules, seem to be required for this phenomenon [23] but several questions about the mechanism of this form of memory remain. One is how inhibitory Ly49 receptors can mediate activation in this context. Another is how cells that are resident in the liver can mediate contact hypersensitivity in the bladder and ear: one possibility is that these NK cells are liver-resident at homeostasis but are able to traffic during an immune response.

It is not yet known whether a similar population of memory NK cells is liver-resident in humans, but there is some evidence that they may be present in macaques [24]. Suggestively, immune-deficient mice reconstituted with human hematopoietic stem cells develop a population of NK cells in their livers that can mediate recall responses to vaccination with HIV-env [25], but whether liver-resident NK cells in healthy humans have this ability remains to be seen.

### 3.2. Cytokine-Induced NK Memory

Stimulation of NK cells with a combination of IL-15, IL-12 and IL-18 leads to enhanced responses to rechallenge, either with cytokines or tumor cells. Adoptively-transferred cytokine-stimulated murine NK cells are more able to produce IFNγ in response to restimulation three weeks later [26] and subsequent experiments showed that the cells can survive for up to three months and mediate in vivo control of lymphoma and melanoma [27]. The same effect was seen in immunodeficient mice reconstituted with human NK cells and challenged with human lymphoma [28] or melanoma [29] cells. Cytokine-stimulated human NK cells maintain their enhanced ability to produce IFNγ in response to restimulation for up to three weeks in vitro [29]. They also retain this ability for at least one week in vivo following transfer to acute myeloid leukemia patients, and a phase I trial suggests that this approach might have some therapeutic benefit [28].

In contrast to the response mediated by liver-resident NK cells, cytokine-driven NK memory responses are not in themselves antigen-specific. However, antigen-specificity resides in CD4^+^ T cells, which make IL-2 required for the response [27,30] Further, it is not clear how long-lived the response is: although the cells persist for up to three months in mice, enhanced responses at this timepoint have not been demonstrated [27] and the ability of cytokine-stimulated human NK cells to produce IFNγ declines over time [29]. Therefore, the cytokine-induced enhancement of NK cell ability to produce IFNγ may not strictly fulfil the definition of immune memory, with some investigators preferring to refer to it as “training” or “priming” [31]. However, cytokine-mediated effects do appear to feed into the antigen-specific phenomenon of NK cell memory to CMV.

### 3.3. CMV-Driven NK Memory

It has long been known that NK cells bearing the activating receptor Ly49H proliferate in response to MCMV infection [32], a phenomenon that arises as a result of these cells recognizing the viral protein m157 [15]. More recently, it has come to light that these cells display hallmarks of memory. Ly49H^+^ NK cells expand on primary MCMV challenge and contract once the infection is controlled, but the remaining cells are long-lived and can re-expand in response to rechallenge [33]. Furthermore, adoptive transfer of these cells to immunodeficient neonatal mice protects against MCMV [33]. The memory phenotype is specific to MCMV: indeed, these memory cells are less responsive to challenge by influenza or Listeria than naïve NK cells [34]. The initial expansion of the cells and their later survival depends on IL-12 [35] and is potentiated by the adhesion molecule DNAM1 [36].

Liver-resident NK cells in mice (or ILC1) are also able to control MCMV infection in an IL-12-dependent manner [37] and have recently been shown to mediate recall responses to the virus [38]. However, in contrast to conventional NK cells, liver-resident NK cells recognize the viral glycoprotein m12 [38], probably via their activating NK1.1 receptors [39].

Evidence for NK cell memory to HCMV comes from the observation that an NKG2C^+^ subset of NK cells expands in acute infection [40,41,42] and remains overrepresented in seroconverted individuals [41,43,44,45]. This contrasts with the situation in mice, in which the Ly49H^+^ subset returns to baseline levels once acute infection is resolved [33]. In addition to NKG2C, these adaptive NK cells characteristically express increased CD2, CD7, CD57, KIRs and LILRB1 and decreased expression of the signaling molecules FcεRγ, SYK and EAT-2 [41,44,45,46,47]. It has been suggested that the reduction in the expression of signaling molecules could reduce tonic signaling, potentiating greater responses on activation [44]. In line with this, adaptive NK cells display increased reactivity to antibody-coated [43,44] and HCMV-infected target cells [44,48] in vitro, and ex vivo during HCMV reactivation [42]. On the other hand, they are less responsive than naïve cells to cytokine stimulation [43,44]. Whether these cells protect against HCMV is not yet clear, but one report that their expansion in an infant lacking T cells led to the clearance of an acute HCMV infection without the need for antiviral drugs is suggestive [49]. 

The expansion of this NKG2C^+^ population of adaptive NK cells is driven by a number of factors. When human NK cells are cultured with HCMV-infected fibroblasts, the NKG2C^+^ population expands in a manner that is dependent on the interaction between NKG2C and its ligand HLA-E [50,51]. Monocyte-derived IL-12 is necessary but not sufficient for expansion [47,51]. However, it is clear that NKG2C is not the only determinant of expansion of adaptive NK cells, since a subset of NK cells that resembles them in all but NKG2C expression expands in individuals homozygous for a deleted allele of NKG2C [46,52]. Examination of NK cells from these individuals demonstrates that signaling through CD2 and CD16 can synergistically drive the expansion of this population [46]. There is also evidence that KIR signaling may be involved, since KIRs are overexpressed on these cells [40,43,44] and in coculture experiments deletion mutants of US2/11, which give a smaller “missing self” signal, were less able to promote the expansion of the population [50]. Although the leader peptide of the viral immune evasion protein UL40 has recently been shown to be a key determinant of NKG2C binding to HLA-E [47], this coculture study failed to find a defect in the ability of UL40 deletion mutants to promote the expansion of the NKG2C^+^ subset, providing further evidence that NKG2C signaling is not necessary for this process.

Following their expansion, the phenotype of the adaptive NK cells is maintained by epigenetic changes in DNA methylation, similar in a number of ways to those seen in CD8^+^ effector T cells, compared to naïve cells [44]. The transcription factor PLZF, which is required for the maintenance of innate and innate-like cells including conventional NK cells, iNKT cells and γδ T cells, is downregulated in adaptive NK cells and it has been proposed that derepresses the adaptive features of these cells [44]. 

## 4. NK Cells and HCMV

HCMV has a complex relationship with NK cells, and the extent to which NK cells are involved in the control of HCMV is a subject of some debate [53]. Clinical situations in which NK cells are defective are associated with increased susceptibility to HCMV [54,55,56] but this is hard to reconcile with reports that NK cells isolated from blood are unable to effectively control replication of wild type HCMV in vitro [55,57]. A possible explanation for this turns on the idea that the immune system as a whole is in a fine balance with HCMV, such that the immune response can control infection (demonstrated by the increased susceptibility among the immunocompromised) but cannot clear the virus: as such it is not clear that the activity of purified NK cells in a culture system necessarily reflects their behavior in vivo. Another possibility is that the major NK cell populations controlling HCMV are tissue-resident, rather than the circulating NK cells used in these experiments. The finding that liver-resident NK cells control MCMV in mice could support this hypothesis [37]. 

Another line of evidence that NK cells are important for the control of HCMV is the large portion of the viral genome that is dedicated to encoding NK cell immune evasion molecules (summarised in Table 1). HCMV is well-known for its ability to evade T cell recognition by encoding proteins that interfere with almost all stages of antigen processing and presentation through MHC-I molecules, but this has the potential to leave the virus vulnerable to “missing self” recognition by NK cells [58]. To avoid this, HCMV encodes MHC-I mimics and proteins that restore or replace MHC-I molecules on the cell surface, thereby engaging NK cell inhibitory receptors and dampening NK cell cytotoxicity. HCMV encodes the MHC-I mimic UL18, which engages the inhibitory receptor LILRB1 [59], as well as UL40, which maintains HLA-E expression on the infected cell surface [47,60]. 

Viral infection also induces cellular stress that could result in upregulation of stress ligands recognized by NK cell activating receptors. Such stress ligands include the UL16 binding proteins (ULBPs), the MHC-I polypeptide-related sequence A/B (MICA/B), nectin and nectin-like proteins (CD112, CD155), CD48 and CD58 [53,58,61,62,63,64,65]. These stress-induced molecules are recognized by the activating receptors NKG2D, DNAM-1, 2B4 and CD2 with NK cell activation typically requiring signaling through more than one of these receptors [66,67]. HCMV counters these stress responses by encoding proteins that downregulate stress-induced ligands on infected cells and in some cases also directly target the corresponding activating receptor [58,68]. However, the dynamic relationship between virus and host is demonstrated by the presence of the highly prevalent MICA*008 allele in humans that appears to have been positively selected. This allele acquired a frameshift mutation to escape downregulation by HCMV UL142, but in response, HCMV has evolved the US9 protein, which targets this ‘escape variant’ highlighting the adaptability of the virus [69].

Other activating receptors targeted by HCMV include the natural cytotoxicity receptor NKp30, with the viral protein pp65 causing dissociation of the receptor from one of its adaptor molecules, CD3ζ [68]. NK cells can also target virally-infected cells by ADCC through the expression of FcγRIII (CD16) that recognizes the Fc portions of IgG. Signaling through FcγRIII alone is sufficient for NK cell activation and cytotoxicity. HCMV encodes several immune evasion molecules that bind the Fcγ chain of IgG and thus prevent activation of NK cells through FcγRIII [66].

## 5. HCMV Vaccination: Current Strategies

The first HCMV vaccine candidates were live attenuated viruses with the laboratory strains AD169 [70] and Towne [71] as vaccines. AD169 was soon dropped from trials due to low efficacy. The Towne strain conferred protection against challenge with a non-attenuated strain but failed to prevent acquisition in later trials and was not taken forward [72]. The next candidates were based on purified recombinant viral glycoproteins, predominantly glycoprotein B (gB) because of its key function in cell-entry and infection [73]. The major immunization studies with gB have given it in combination with different adjuvants, most notably MF59 and AS01 that had been extensively tested in a number of human and animal trials. The gB/MF59 vaccine offered partial protection, eliciting anti-gB antibodies and reducing HCMV acquisition in two phase II trials [74,75] and reducing virologic parameters in HCMV-negative transplant patients who received an organ from an HCMV-positive donor [76]. The gB AS01 vaccine formulation has shown promising results in guinea pig models of CMV congenital infection [77] and robust antibody response with evidence of virus neutralization in a phase I study in humans [78]. However, no further results have been published and the vaccine did not proceed to further testing. Although gB/MF59 did not reach significant efficacy to support recommendation and licensure, approaches using gB in combination with adjuvants have been one of the most successful so far and underpins the development of novel candidates currently under clinical testing. 

Studies investigating the role of other HCMV glycoproteins in infection, and characterizing immune responses to HCMV, supported the inclusion of other glycoproteins in vaccine candidates hoping to increase the breadth of efficacy of vaccines. For instance, the pentameric complex (comprising gH/gL/pUL128L/UL130/UL131) has been shown to be indispensable for infection of epithelial and endothelial cells [103] and is considered an important target for vaccine induced neutralizing antibody responses [104]. Paradoxically, the pentamer is also important for cell to cell spread of the virus which impede the action of neutralizing antibodies [105]. The detection of antibodies against the pentamer have been reported as a correlate of protection with reduced congenital transmission upon primary infection, but not in seropositive mothers. Furthermore, infusion of pentamer and gH antibodies resulted in lower levels of HCMV reactivation in kidney transplant recipients [106,107,108]. As a result, pentameric complex glycoproteins have been included in a number of candidates either alone or in association with gB. 

However, approaches relying on recombinant proteins encounter several hurdles for purification and manufacturing, limiting the number and type of antigens that can be included in the vaccine [109]. To circumvent such issues, viral vectors and self-replicating nucleic acid platforms are being trialed and demonstrating good immunogenicity levels. Such approaches can include more antigens in constructs without increasing the complexity of the manufacturing process and holds great promise in the vaccine field. A number of other candidates, harnessing the entire breadth of adjuvant and vaccine strategies, are being tested against HCMV [10].

### 5.1. Challenges of HCMV Vaccine Development

Several characteristics of HCMV may be contributing to the slow development of a vaccine. HCMV pathogenesis presents as a varied array of disease manifestations and the complex host-pathogen interaction shaped by years of co-evolution has led to a virus with multiple immune evasion mechanisms, posing a great challenge for vaccine design. 

HCMV pathogenesis is generally associated with primary infection, intrauterine transmission [110], and infection or reactivation in immunosuppressed individuals, with HCMV-negative transplant patients receiving a solid organ from an HCMV-positive donor [111] and, conversely, HCMV-positive patients receiving a stem cell transplant from a HCMV seronegative donor at the greatest risk [7]. To effectively reduce the burden of disease, an ideal vaccine would prevent virus acquisition in seronegative individuals, and viral shedding, systemic replication and related pathogenesis, and vertical transmission in seropositive. However, the current evidence in the field suggests that the type of immune response required to prevent acquisition, pathogenesis and transmission are distinct in terms of the antigens targeted and the type of response required. In order to address such issues, an HCMV vaccine is likely to require several antigens and potent adjuvants that can induce activation of a range of immune cells to support the establishment of memory. 

Mounting evidence points for the need of a multi-antigen vaccine and all the candidates currently in clinical trials include more than one antigen, most commonly gB and components of the pentameric complex alongside the immunodominant T cell epitopes IE1 and pp65 [112,113]. Such an approach is likely to increase the breadth and efficacy of the vaccines by interfering with the action of a wider range of targets underpinning infection of different cells types and pathways of disease. Additionally, responses by a more diverse repertoire of polyfunctional HCMV-specific T cells correlate with reduced viremia and systemic spread of HCMV [114], so this approach is likely to improve efficacy. Immunodominance also underpins the choice of antigens for induction of humoral immune responses. However, it is possible that epitopes can induce strong responses that are not necessarily protective and thus reduce the efficacy of a vaccine. This has been suggested for gB [115] in which non-neutralizing domains such as antigenic domain 1 (AD1) are highly immunogenic and have been hypothesized toact as a decoy epitope [116], hampering the response against neutralizing domains that are correlated with better outcome such as AD2 [117,118]. 

An effective response against the various stages in the life cycle of an HCMV infection and in the different subgroups of hosts is likely to require activation of various arms of the immune system. For instance, orchestrated induction of strong mucosal immunity, multifunctional circulating antibodies with neutralizing and non-neutralizing functions, and robust innate activation to support the development of effective memory responses may be key to the control of HCMV. However, inactivated and subunit vaccines have reduced ability to engage and induce all the immune components that are usually induced by natural infection and pathogen-associated signals, and the response will vary according to the type of adjuvant or delivery platform, and route of administration. Identifying safer strategies for live-attenuated vaccines or adjuvants that provide strong and varied signal to immune cells, such as pathogen-associated molecular patterns (PAMPs) that can engage several immune cells, may be the key to achieving this.

### 5.2. Current Candidates

Even in face of all the aforementioned hurdles, advances in basic virology, immunology of vaccines, and novel strategies for antigen delivery offer hope in the long quest for an HCMV vaccine. Currently, at least 15 vaccines in advanced stage of development [10], of those 14 are inactivated or subunit vaccines and 1 is a live attenuated vaccine. The live attenuated vaccine, Merck V160, is based on the Merck AD169 strain with a restored pentameric complex and includes a molecular shield that renders the virus replication-defective and offers enhanced safety. Results from phase 1 studies demonstrated induction of and high frequency of memory B cells to similar levels to those induced by natural infection without shedding of virus in urine and saliva [119] and the vaccine is now undergoing phase II efficacy trials. Of the inactivated and subunit vaccines, the candidate that is most advanced in clinical trials is a self-replicating RNA-based vaccine containing six mRNAs coding for the components of the pentameric complex and gB. The vaccine induced robust antibody responses that can neutralize infection of both fibroblasts and epithelial cells, as well as a substantial multi-antigen cellular response [120]. Positive preliminary results from an ongoing phase II clinical trials in women of childbearing age prompted the beginning of recruitment for a phase III trial.

## 6. NK Cells in HCMV Vaccination

**Figure 1:**
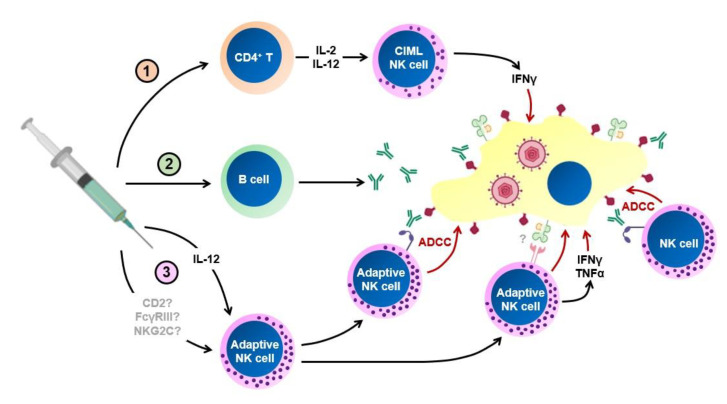
Mechanisms by which NK cells could be harnessed in human cytomegalovirus (HCMV) vaccination. 1,2. Vaccination traditionally targets aspects of the adaptive immune response, for which NK cells can act as effectors. 1. Antigen-specific CD4^+^ T cells produce IL-2 and IL-12, which can lead to the production of cytokine-induced memory-like NK cells (CIML). These produce increased levels of IFNγ, which could help control HCMV. 2. B cell produce antibodies, which can coat virally-infected target cells. NK cells kill the antibody-coated target cell by ADCC, controlling the virus. 3. Natural HCMV infection and some vaccine formulations may be able to induce adaptive NK cells via the induction of IL-12. As-yet-undefined receptor-ligand interactions are also likely to be important for the induction of these cells, with CD2, FcγRIII (CD16) and NKG2C key candidate molecules to be involved in this process. Adaptive NK cells display increased ability to mediate ADCC, and to release cytotoxic granules in response to HCMV-infected cells, although the receptor-ligand interactions that are required for this are not yet fully defined. They may further be able to control the virus by their increased ability to produce IFNγ and TNFα.

Thus far, approaches to HCMV vaccination have focused on raising adaptive immune responses to specific antigens, particularly humoral responses against the pentameric complex and gB or T cell mediated responses against IE1 and pp65. However, given the evidence that NK cells can mediate protection and memory responses against CMVs, we propose that it is now worth considering whether it will be possible to harness these responses to improve vaccine efficacy. There are three ways in which NK cells can act to improve vaccine responses (Figure 1). The first two of these involve the interaction between NK cells and components of the adaptive immune response, with NK cells either supporting adaptive responses, or acting as effectors of the antibody response via ADCC. The third requires an attempt to generate memory responses within the NK cell compartment itself. A combination of all three approaches may be the most effective. Indeed, live attenuated vaccine candidates are likely to stimulate all three arms of the NK cell response, in addition to adaptive responses.

### 6.1. NK Cell Support of Adaptive Responses

The indirect role of NK cells in shaping protective responses induced by vaccines has been demonstrated in animal models and humans in a number of studies employing different modalities of vaccines. NK cells produce IFNγ in response to rabies, influenza and DTP vaccinations, with increased levels of NK cell responsiveness observed as much as six months following vaccination [121,122,123]. This is likely to boost vaccine efficacy since vaccine-induced NK activation and cytokine production in response to influenza vaccination recruited IL-6-producing dendritic cells (DCs) to the lymph nodes, that supported anti-Influenza B cell responses. Depletion of either IFNγ or IL-6 resulted in reduced frequency of influenza-specific antibody secreting cells [124]. 

Recently, NK cells were suggested to contribute to the early protective response of the Ebolavirus vaccine rVSV-ZEBOV [125]. System vaccinology studies identified the post-vaccination phenotype of NK cells to be positively correlated with antigen-specific responses induced by the vaccine on day 28 [126]. The vaccine induced several NK cell activating factors, including IL-15 and tumor necrosis factor-α (TNFα) and resulted in higher expression of CXCR6, NKG2D, and CD56. These results not only show that NK cells are activated in the early stages in response to inactivated and live attenuated vaccines, but also that frequency, phenotype, and cytokines produced by NK cells correlates to more effective humoral responses. 

### 6.2. NK Cells as Effectors of the Antibody Response

Clinical isolates of HCMV usually spread as cell-associated, rather than cell-free, virus and in such situations neutralizing antibodies may not be particularly protective whereas ADCC, which targets infected cells, is more likely to be effective at controlling the virus [105]. Key to success is raising an appropriate IgG response to a viral protein expressed on the surface of infected cells. Vaccine strategies that harness ADCC have been trialed for cell-associated viruses such as HIV. IgG1 and IgG3 are the IgG subclass most able to promote ADCC [127] and participants who raise an IgG3 response against the exposed loops of HIV-env display greater protection against later infection [128]. Similarly, levels of IgG raised against Ebolavirus glycoprotein by a prime-boost vaccine strategy correlated with NK cell activation [129]. A similar approach could be considered for HCMV vaccination and this is particularly attractive in the light of findings that HCMV-driven adaptive NK cells, if they can be induced, express high levels of FcγRIII (CD16) and are extremely effective mediators of ADCC [43,44]. Another platform that could directly harness the effector function of NK cells through vaccination are Fc-fusion proteins. The Fc effector domain of immunoconjugates can directly engage activating FcγRIII in NK cells and enhance ADCC against specific targets as demonstrated in an immunization model against cancer [130].

### 6.3. Induction of NK Cell Memory

The memory phenotype in NK cells can be induced either by receptor-ligand interaction or by cytokines. For viruses where receptor-ligand responses had been demonstrated, such as CMV and Influenza A, both mechanisms can be harnessed by vaccination. In mice, CMV-driven adaptive NK cell responses protect against later infection [33] and there is some evidence that the same is true in humans [49]. If this is the case, vaccine strategies that induce such adaptive NK cells could provide an extra level of protection, compared to those that induce only T and B cell responses, and could also synergize with these responses via the increased ability of these NK cells to mediate ADCC. 

Following influenza vaccination, although the majority of the antigen specificity of NK cell responses is found in the CD4^+^ T cell compartment [27,30,121], there is some evidence that broad memory responses can be mediated by the ability of NK cells to recognize viral haemagglutinin via NKp46 [131], highlighting the possibility that NK cell memory to HCMV could also be mediated by an activating NK cell receptor. Which NK cell receptors and ligands drive the expansion of adaptive NK cells in response to HCMV, and which are required to mediate responses, is not yet clear, but studies on adaptive NK cells from NKG2C-null individuals suggest that CD2 and FcγIII are leading candidates [46]. Future work in which the roles of these and other molecules in the production and function of adaptive NK cells are fully defined may allow us to design vaccines that specifically induce these cells, providing greater protection against HCMV. However, there are several barriers to our ability to achieve adaptive NK cell responses in the context of vaccination, not least that we do not yet fully understand the mechanisms by which HCMV drives the expansion of these cells. Certainly, IL-12 is key player in the process [47,51] so vaccination strategies that induce IL-12 are likely to be useful in this endeavor. 

NK responses have been reported in vaccines employing live attenuated virus [131,132] viral vectors [125] and non-infectious virus-like particles [133]. All three strategies have in common the provision of a number of PAMPs that are strong activators of pattern recognition receptors (PRRs) present in immune cells. The strategy of providing robust activation of PRRs through PAMPs as adjuvants of vaccines has been extensively demonstrated with a major focus on DCs and macrophages [134,135], and the finding that monocyte-derived IL-12 [47,51] is important for the induction of adaptive NK cells suggests that this approach could be harnessed in the context of NK cells. Further studies investigating adjuvant and vaccine formulation that can optimally engage NK cells that are likely to enhance efficacy of responses and can be crucial for the development of a HCMV vaccine. In mice, TLR ligands such as CBLB502 (TLR5 agonist), imiquimod and guasiquimod (TLR7/8) ligand and CpG (TLR9 ligand), have been shown to enhance NK cell effector function against tumors [136,137]. Nanoparticles containing TLR ligands such as CpGs induce IFNα and IL-12 and have been extensively harnessed as adjuvant in a number of vaccine candidates: such a strategy can be used to induce NK cells. Adjuvants that activate NK cells and induce IL-12 production may result in both adaptive [47,51] and cytokine-induced memory-like NK cells [26,28,29].

## 7. Conclusions

The immune response to natural HCMV infection is broad and complex. Clearly, this response is effective at limiting HCMV pathogenesis but is not sufficient to prevent the establishment of lifelong latency, to promote the eradication of latent virus nor completely prevent the risk of reinfection. The ability of HCMV to persist in the face of such an immune response is underpinned by the prodigious immune evasion mechanisms encoded by the virus. The challenge moving forward is to understand the elements of the immune response to HCMV that make the greatest contribution to its control in vivo and identify ways to harness this with vaccination. Logically, these approaches seek to educate the adaptive immune response and will likely form a major part of our attempts to control HCMV. 

It is becoming increasingly clear that NK cells, classically considered innate effectors, have traits associated with adaptive responses—particularly against CMV infection. Furthermore, they are often enriched in tissues which are important sites of HCMV replication, such as the liver and pregnant uterus. In light of the multitude of NK immune evasion genes expressed by HCMV, these findings suggest they could play an important role in control of infection. Indeed, it is the prevention of vertical transmission that is a major driver of HCMV vaccine research. An important caveat of this will be understanding the impact of HCMV infection on the NK cell repertoire if vaccination of both HCMV seropositive and seronegative individuals is the goal: will it be possible to evoke the same responses in seropositive individuals in which adaptive NK cells have already been established? Furthermore, HCMV seropositivity is itself known to alter NK cell responses to vaccination [138] and this may impact the effectiveness of NK cell-based vaccine strategies in HCMV seropositive individuals.

Identification of the key characteristics of an effective NK response against HCMV coupled with intervention strategies that can enhance these responses could potentially enrich ongoing efforts to design an effective vaccine against this clinically important pathogen.

## Figures and Tables

**Table 1:** Mechanisms by which HCMV evades NK recognition.

Receptor Type	Receptor	Ligand	HCMV Immune Evasin	Evasion Mechanism
Activating	Activating KIR	MHC-Ia	US2, US3, US6, US11	Reduction in MHC-I surface expression; US2, US11- proteasomal degradation [79,80,81]; US3- ER retention of MHC-I [82]; US6- blocks TAP mediated peptide translocation [83].
	-	HLA-G	US2, US10	Reduction in HLA-G surface expression; US2- proteasomal degradation [79,80,81]; US10- degradation of HLA-G [84].
	CD94-NKG2C/E/H	HLA-E	US6, UL40, miR376a	UL40 encodes a TAP-independent signal peptide that stabilises HLA-E surface expression [85]. miR376a blocks HLA-E surface expression [86]. US6 blocks TAP dependent peptide translocation [83].
	NKp30	B7-H6	US18, US20, pp65 (UL83)	US18, US20, Lysosomal degradation of activation receptor ligands [87].
	-	BAT-3	pp65 (UL83)	pp65-binds NKp30 causing CD3***ζ*** dissociation [68].
	NKG2D	MICA/B	UL16, UL142, UL148A, US9, US18, US20, miR-UL112	UL16, UL142- intracellular retention [88,89]; UL148A, US18, US20- lysosomal degradation [87,90]; US9- proteasomal degradation (MICA*008) [69], miR-UL112- downregulation of MICB expression [91].
	-	ULBPs	UL16, UL142, US12, US13, US20	UL16, UL142- intracellular retention [88,92,93]; US20- lysosomal degradation [87]; US12, US13- downregulation of ULBPs [94].
	DNAM-1	CD112	UL141 (requires US2)	ER retention [95].
	-	CD155	UL141	ER retention [96].
	TACTILE	CD111	-	-
	-	CD155	UL141	ER retention [96].
	2B4	CD48	-	-
	CD2	LFA-3	UL148	Lysosomal degradation [97].
	CD16	Fc of IgG	RL11-13, UL119-UL118	Fc*γ* binding and inhibition of Fc receptor signalling, inhibition of ADCC [98].
	TRAIL	TRAIL-R1/-R2	UL141	ER retention [99].
	CD45	pUL11	pUL11	Inhibition of CD45 mediated signalling through direct binding of pUL11 [100].
Inhibitory	Inhibitory KIR	MHC-I polymorphisms	-	-
	LIR-1	MHC-Ia	UL18	MHC-I homolog [101].
	CD94-NKG2A/B	HLA-E	UL40	UL40 encodes a TAP-independent signal peptide that stabilises HLA-E surface expression [85].
	TACTILE	CD111	-	-
	-	CD155	UL141	ER retention [96].
	TIGIT/ PVRIG	CD112	UL141 (requires US2)	ER retention [95].
	-	CD155	UL141	ER retention [96].
	Cadherins	KLRG1	-	-
	LLT1	CD161 (NKR-P1A	-	-
	-	WAVE2/F-actin	UL135	Suppression of immune synapse formation [102].

BAT3: HLA-B associated transcript 3; DNAM-1: DNAX accessory molecule-1; HLA: human leukocyte antigen; KIR: killer-cell immunoglobulin-like receptors; KLRG1: killer cell lectin-like receptor subfamily G member 1; LFA3: lymphocyte function-associated antigen 3 (CD58); LIR: leukocyte immunoglobulin-like receptor; LLT1: lectin-like transcript-1 (CLEC2D); MICA/B: MHC class I polypeptide–related sequence A/B; miR: microRNA; NKR-P1: natural killer cell receptor protein 1 (KLRB1-killer cell lectin-like receptor subfamily B, member 1, CD161); PVRIG: poliovirus receptor related immunoglobulin domain containing; TACTILE: T cell activation, increased late expression (CD96); TIGIT: T cell immunoreceptor with Ig and ITIM domains; TRAIL: TNF-related apoptosis-inducing ligand; ULBP: UL16 binding protein; US/L: unique short/long; WAVE2: Wiskott-Aldrich syndrome protein family member 2.
